# Multimodal skin imaging in chromoblastomycosis: Diagnostic correlation and treatment monitoring during adjunctive fractional carbon dioxide laser therapy

**DOI:** 10.1016/j.jdcr.2026.05.059

**Published:** 2026-07-01

**Authors:** Hanhui Nie, Zhenmou Xie, Yiting Fu, Quanhui Zheng, Hongfang Liu, Liyan Xi, Yangxia Chen, Yinghui Liu

**Affiliations:** a Department of Dermatology, Dermatology Hospital, Southern Medical University, Guangzhou, China; b Department of Clinical Laboratory, Dermatology Hospital, Southern Medical University, Guangzhou, China; c Department of Ultrasound, Dermatology Hospital, Southern Medical University, Guangzhou, China; d Department of Dermatology, Sun Yat-Sen Memorial Hospital, Sun Yat-Sen University, Guangzhou, China

**Keywords:** antifungal therapy, chromoblastomycosis, dermoscopy, fractional carbon dioxide laser, high-frequency ultrasound, reflectance confocal microscopy

## Introduction

Chromoblastomycosis (CBM) is a chronic implantation mycosis caused by melanized fungi and is often characterized by delayed diagnosis, long disease duration, and incomplete response to therapy.^1^^,^^2^ The diagnosis is usually established by direct microscopy, fungal culture, and histopathology; however, noninvasive imaging can provide rapid bedside clues and may help monitor treatment response over time.^3^, ^4^, ^5^, ^6^, ^7^ We report a case of long-standing CBM caused by *Fonsecaea pedrosoi* in which dermoscopy, reflectance confocal microscopy (RCM), and high-frequency ultrasound were used alongside conventional testing for diagnostic correlation and serial follow-up during combined therapy.

## Case report

A 55-year-old man who worked as a farmer presented with a 30-year history of progressive plaques on the right lower extremity. The eruption began as a purplish-red plaque below the knee and gradually extended to the thigh, calf, and popliteal fossa. He had previously been diagnosed elsewhere with cutaneous tuberculosis and had received isoniazid, 0.3 g once daily, rifampin, 0.45 g once daily, ethambutol hydrochloride, 0.75 g once daily, and vitamin B6 for ∼3 years without meaningful improvement. He reported no history of diabetes or other known immunocompromizing disease. Baseline evaluation during our admission did not reveal an obvious immunocompromizing condition: complete blood count showed no leukopenia or lymphopenia; serum glucose and immunoglobulin levels were within reference ranges; human immunodeficiency virus serology, antinuclear antibody, T-Spot test for tuberculosis, and interferon-gamma release assay results were negative; and T helper 1/T helper 2-related cytokines showed no obvious abnormalities. On examination, there were extensive verrucous and infiltrated purplish-red plaques with yellow-white crusting and scaling on the lower portion of the right limb (Fig 1).

**Fig 1:**
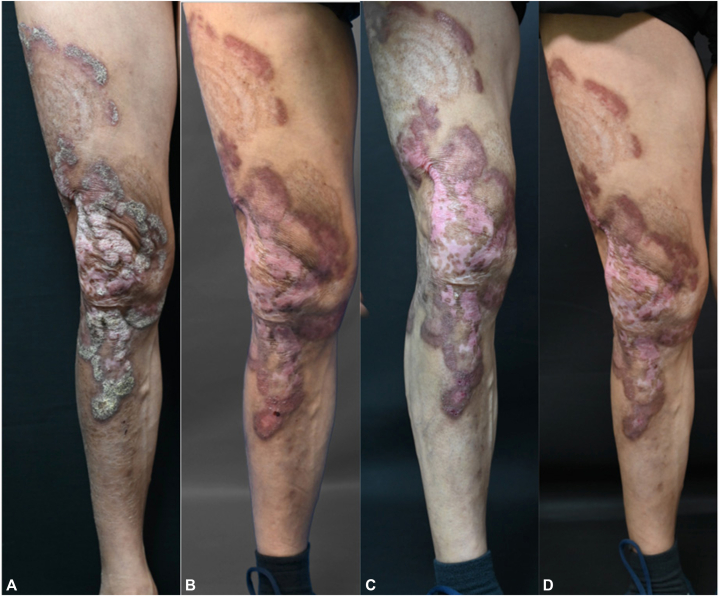
Chromoblastomycosis. Clinical photographs of the right lower extremity. Baseline examination shows extensive verrucous and infiltrated plaques with crusting and scale **(A)**. Serial follow-up demonstrates progressive flattening and decreased erythema after combined fractional carbon dioxide laser therapy and oral itraconazole plus terbinafine at ∼1 month **(B)**, 2 months **(C)**, and 3 months **(D)**.

Direct microscopy of skin scrapings treated with 10% potassium hydroxide showed thick-walled brown muriform cells (Fig 2, *A*). Fungal culture on Sabouraud dextrose agar incubated at 25 °C for 14 days produced black filamentous colonies (Fig 2, *B*), and lactophenol cotton blue staining demonstrated septate hyphae with dark conidia (Fig 2, *C*). Internal transcribed spacer sequencing identified *F pedrosoi*. Histopathologic examination showed marked epidermal hyperplasia and a mixed inflammatory infiltrate with multinucleated giant cells (Fig 2, *D*). Periodic acid–Schiff/D-PAS staining demonstrated fungal spores, whereas acid-fast staining was negative. Because of the chronicity and previous diagnosis of cutaneous tuberculosis, additional studies were reviewed for possible coexisting infection: bacterial culture showed no pathogenic growth, and fungal culture from biopsy tissue later yielded a dematiaceous fungus. Together with the mycologic findings, these results supported the diagnosis of CBM and did not suggest another coexisting infectious process.

**Fig 2:**
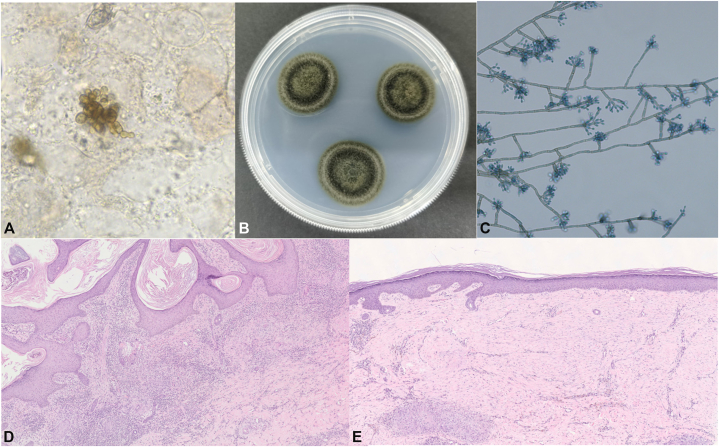
Chromoblastomycosis. Diagnostic mycology and histopathology. Direct microscopy of skin scrapings demonstrates muriform cells **(A)**. Fungal culture on Sabouraud dextrose agar shows black filamentous colonies after 14 days of incubation at 25 °C **(B)**. Lactophenol cotton blue preparation demonstrates pigmented septate hyphae and conidia **(C)**. Histopathologic sections show epidermal hyperplasia and a mixed inflammatory infiltrate before treatment **(D)** and reduced inflammation with increased fibrosis/scar tissue after treatment **(E)**. **(D** and **E,** Hematoxylin-eosin stain; original magnifications: **D,** ×50; **E,** ×50.**)**

Baseline dermoscopy showed small black dots, yellow-orange structureless areas, and overlying scales/crusts, findings that have been associated with transepidermal elimination and granulomatous inflammation in CBM (Fig 3, *A*).^3^, ^4^, ^5^ RCM demonstrated multiple hyperreflective round bodies measuring ∼5 to 12 μm, corresponding to the size range reported for muriform cells (Fig 3, *C*).^6^^,^^7^ High-frequency ultrasound showed marked epidermal thickening and heterogeneous hypoechoic change within the papillary dermis, with a maximal thickness of 4.4 mm (Fig 4, *A*).

**Fig 3:**
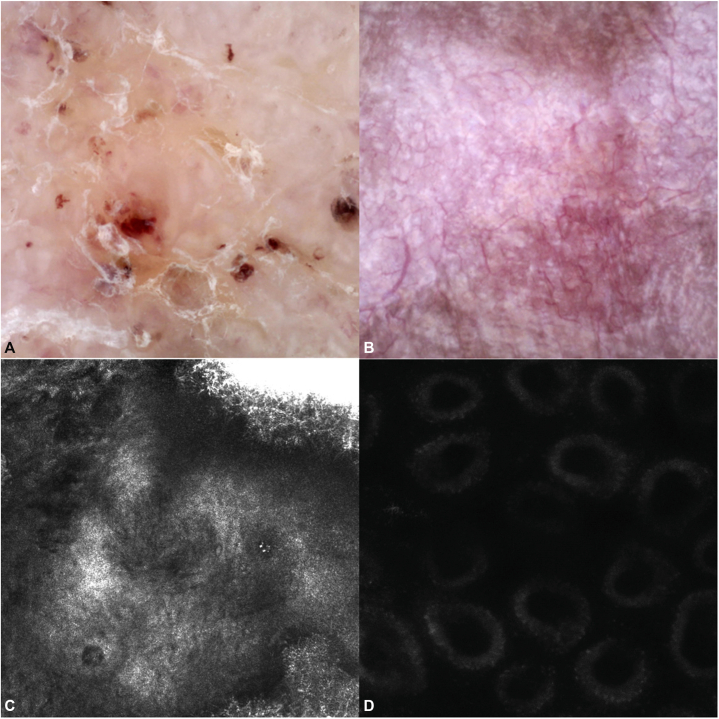
Chromoblastomycosis. Dermoscopy and reflectance confocal microscopy. Dermoscopy at baseline shows *black* dots, scales/crusts, and *yellow-orange* structureless areas **(A**, *left***)**; after treatment, the *black* dots and *yellow-orange* areas have resolved with residual scar-like change **(B**, *right***)**. Reflectance confocal microscopy shows hyperreflective round bodies at baseline **(C**, *left*, *arrow***)** and absence of these structures after treatment **(D**, *right***)**.

**Fig 4:**
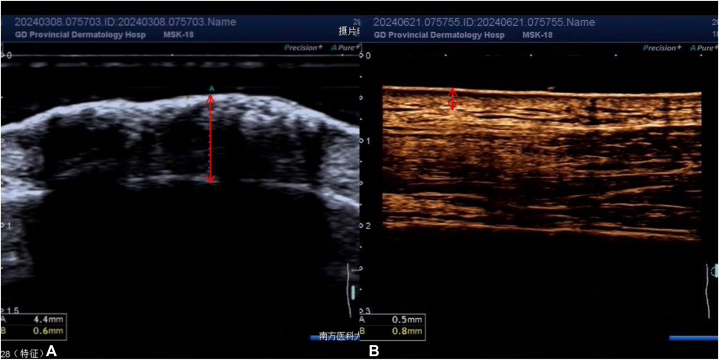
Chromoblastomycosis. High-frequency ultrasound. **A,** Baseline imaging demonstrates marked epidermal thickening and superficial dermal hypoechoic change with maximal thickness of 4.4 mm. **B,** Follow-up imaging after treatment shows marked reduction in thickness to 0.8 mm and improvement in dermal echotexture.

Antifungal susceptibility testing suggested lower minimum inhibitory concentrations for terbinafine and voriconazole than for itraconazole and posaconazole. In view of the need for prolonged outpatient treatment, we selected oral terbinafine together with itraconazole rather than voriconazole because terbinafine also showed favorable in vitro activity and itraconazole remains a commonly used, accessible agent for CBM in routine practice. The patient was treated with monthly fractional carbon dioxide laser sessions together with oral itraconazole, 200 mg daily, and terbinafine, 250 mg daily. After 3 months, the plaques had flattened substantially, with decreased crusting and erythema (Fig 1). Follow-up dermoscopy showed resolution of black dots and yellow-orange structure areas, leaving residual scar-like and vascular changes (Fig 3, *B*). RCM no longer showed the previously noted hyperreflective round bodies (Fig 3, *D*). Ultrasound demonstrated marked reduction in epidermal thickness to 0.8 mm and improvement of the prior hypoechoic dermal changes (Fig 4, *B*). Repeat histopathology showed decreased inflammatory infiltrates and increased fibrosis/scar tissue (Fig 2, *E*). No pruritus, burning sensations, or laboratory abnormalities were observed during treatment.

## Discussion

This case illustrates 2 practical points. First, multimodal skin imaging can complement, but not replace, conventional evaluation in CBM. In routine practice, clinical examination, dermoscopy, direct mycology, and histopathology remain the mainstays of diagnosis and treatment monitoring, whereas RCM and high-frequency ultrasound may serve as adjunctive tools in centers where they are available. Dermoscopy rapidly highlighted black dots and yellow-orange areas; RCM provided a noninvasive correlate for muriform bodies; and ultrasound supplied an objective structural measure that changed in parallel with clinical improvement. Prior reports have already described dermoscopy, in vivo confocal microscopy, and multimodal imaging in CBM.^3^, ^4^, ^5^, ^6^, ^7^ The value of the present case is the clinicopathologic correlation and longitudinal documentation of imaging changes across treatment.

Second, adjunctive fractional carbon dioxide laser therapy may be useful in selected hyperkeratotic or fibrotic lesions, in which drug penetration is thought to be limited. Earlier reports suggest that laser-based approaches may serve as adjuvant therapy in refractory CBM or other difficult fungal infections.^8^, ^9^, ^10^ In our patient, combined laser treatment and oral antifungal therapy was associated with early clinical and imaging improvement, although the relative contribution of each component cannot be determined from a single case.

The main limitations are the single-patient design, short follow-up, and the limited availability of RCM and high-frequency ultrasound across institutions. Nevertheless, this case supports the use of dermoscopy, RCM, and high-frequency ultrasound as complementary tools for diagnosis and response assessment in CBM, particularly when repeated invasive sampling is undesirable, and such modalities are locally available.

### Declaration of generative AI and AI-assisted technologies in the writing process

During the preparation of this work, the authors used ChatGPT (OpenAI) in order to assist with language polishing, sentence restructuring, and improvement of overall clarity. After using this tool, the authors reviewed and edited the content as needed and take full responsibility for the content of the publication.

## Conflicts of interest

None disclosed.
